# Visits to Private Asylums

**Published:** 1903-03-14

**Authors:** 


### VISITS TO PRIVATE ASYLUMS.

By Our Rambling Reporter.

### NORTHWOODS, WINTERBOURNE, NEAR BRISTOL.

Many of our licensed houses have been converted from old mansions into asylums, and however carefully such conversion may have been carried out, and however skilfully the additions may have been made, it is very difficult to ensure that the component parts will hang properly together after conversion, and satisfactorily fulfil their respective purposes. Northwoods has the enormous advantage of having been planned for an asylum, and of having been carried out under the superintendency of a medical man who was long familiar with the requirements of such an institution. It was built about 70 years since, and is a fine specimen of modern classic architecture. Other styles may be more picturesque, but it is doubtful whether any will lend itself better to the arrangements and requirements of a large private asylum. The license dates from the completion of the building, so that it is no parvenu, and it can boast of a long period of success in the treatment of mental diseases.

The asylum can accommodate 50 patients, and each patient has a separate room. Private sitting-rooms can also be arranged for, so that a patient can have as much privacy as is desirable, or may mix with his fellow-patients when he likes. All the day-rooms and bedrooms are nicely furnished, and have a comfortable home-like appearance. The means for both indoor and outdoor amusements are good, and it is evident at a glance that the patients are in every way well cared for. The resident medical officers are Dr. Reginald Eager and Dr. Wilson Eager, both men of great and varied experience in the treatment of the insane. The proportion of nurses to patients is about one of the former to three of the latter.

Verandahs, supported by rows of columns, run along the south fronts of the east and west wings, and from these verandas the airing-courts are reached. They are very prettily laid out, and much more resemble gardens than airing-courts. Those at the extreme ends of the building are surrounded by forest trees, which give the patients much shelter from the sun in summer and from the cold winds during the winter months. But many of the patients are allowed to wander beyond the courts, and as there are 50 acres of land belonging to the asylum, and as much of it is laid out in shrubberies and shady walks, there ought not to be any monotony in the out-of-door exercise.

The asylum faces the south, and looks out on the Lansdowne Hills. Bath lies about 16 miles off by road. Northwoods is 8 miles from Bristol, 5 miles from Yate, and 3½ miles from Patchway. It is expected that a new line will soon be opened which will bring the asylum still nearer to a station.

The scale of payments, of course, varies according to the accommodation required for the patient. Cases are received as low as £165 a year, and some pay as much as £600 a year.

